# Thyroid-Stimulating Hormone Receptor Antibodies in Pregnancy: Clinical Relevance

**DOI:** 10.3389/fendo.2017.00137

**Published:** 2017-06-30

**Authors:** Ines Bucci, Cesidio Giuliani, Giorgio Napolitano

**Affiliations:** ^1^Unit of Endocrinology, Department of Medicine and Sciences of Aging, Ce.S.I.-Me.T., University of Chieti-Pescara, Chieti, Italy

**Keywords:** thyroid-stimulating hormone receptor antibodies, Graves’ disease, pregnancy, fetal hyperthyroidism, neonatal hyperthyroidism

## Abstract

Graves’ disease is the most common cause of thyrotoxicosis in women of childbearing age. Approximately 1% of pregnant women been treated before, or are being treated during pregnancy for Graves’ hyperthyroidism. In pregnancy, as in not pregnant state, thyroid-stimulating hormone (TSH) receptor (TSHR) antibodies (TRAbs) are the pathogenetic hallmark of Graves’ disease. TRAbs are heterogeneous for molecular and functional properties and are subdivided into activating (TSAbs), blocking (TBAbs), or neutral (N-TRAbs) depending on their effect on TSHR. The typical clinical features of Graves’ disease (goiter, hyperthyroidism, ophthalmopathy, dermopathy) occur when TSAbs predominate. Graves’ disease shows some peculiarities in pregnancy. The TRAbs disturb the maternal as well as the fetal thyroid function given their ability to cross the placental barrier. The pregnancy-related immunosuppression reduces the levels of TRAbs in most cases although they persist in women with active disease as well as in women who received definitive therapy (radioiodine or surgery) before pregnancy. Changes of functional properties from stimulating to blocking the TSHR could occur during gestation. Drug therapy is the treatment of choice for hyperthyroidism during gestation. Antithyroid drugs also cross the placenta and therefore decrease both the maternal and the fetal thyroid hormone production. The management of Graves’ disease in pregnancy should be aimed at maintaining euthyroidism in the mother as well as in the fetus. Maternal and fetal thyroid dysfunction (hyperthyroidism as well as hypothyroidism) are in fact associated with several morbidities. Monitoring of the maternal thyroid function, TRAbs measurement, and fetal surveillance are the mainstay for the management of Graves’ disease in pregnancy. This review summarizes the biochemical, immunological, and therapeutic aspects of Graves’ disease in pregnancy focusing on the role of the TRAbs in maternal and fetal function.

## Introduction

Pregnancy represents a challenge to the maternal thyroid gland: the various hormonal variations and the increased metabolic demands occurring during gestation deeply affect thyroid function. This means that several changes in the thyroid hormone production and metabolism are expected during gestation (1). During the first trimester, the human chorionic gonadotropin (HCG) hormone, which shares some structural homologies with thyroid-stimulating hormone (TSH), acts as a thyrotropic agonist, overriding the normal action of the hypothalamic–pituitary–thyroid feedback system. The result of this is a transient increase in free thyroxine (FT4) and a transient reduction in TSH whose plasma concentrations are inversely related to those of the HCG. Due to the high levels of estrogens, the serum thyroxine-binding globulin (TBG) concentration rises almost twofold during the first 20 weeks of gestation and remains at a high level until delivery. This means both serum total thyroxine (T4) and triiodothyronine (T3) concentrations increase while their respective free fractions (FT4, FT3) decrease as low as 10–15%. As a consequence, the pituitary increase secretion of TSH whose concentrations, following the first trimester, return steadily to the normal range and show a slight trend toward an increase in response to the decreased serum-free thyroid hormone levels (2). A new equilibrium is reached with an increase in thyroid hormone production of approximately 50% by the maternal thyroid. In order to achieve greater thyroid hormone production, a higher iodine intake is needed in pregnant women due to a pregnancy-related increase in renal excretion and fetal iodine requirement (3). Pregnancy-related changes in thyroid physiology lead to changes in the thyroid function tests, and therefore, parameters of healthy pregnant women differ from those of euthyroid non-pregnant women. The trimester specific range for TSH, as defined in populations with optimal iodine intake, need to be applied while the interpretation of FT4 values necessitates trimester and method-specific ranges given a significant method-dependent variation in the FT4 measurement in pregnancy (4, 5). In summary, the maternal thyroid gland is designed to increase the thyroid hormone secretion and this could be achieved when the gland is both anatomically and functionally intact as well as the iodine intake being at an adequate level (2). Maternal thyroid hormones play an important role in fetal brain development and because the fetal thyroid produces thyroid hormone starting from week 10–12 of gestation and complete maturation of the hypothalamic–pituitary–thyroid axis is reached at week 20, the fetal development depends on the maternal thyroid for the first half of a pregnancy (6). Thyroid diseases are common in pregnancy and uncontrolled thyroid dysfunction (both overt hypothyroidism and overt hyperthyroidism) is associated with infertility, pregnancy loss, and maternal and fetal/neonatal complications (7). Consequently, the diagnosis and management of thyroid disease in women during preconception, pregnancy, and the postpartum (PP) period is the subject of major attention of scientific associations. Several guidelines have been published and very recently updated (8, 9). Most thyroid diseases affecting childbearing women are autoimmune and up to 20% of pregnant women screened during the first trimester of gestation had positive thyroid autoantibodies (10). Thyroid autoimmunity is associated with infertility as well as with different pregnancy complications such as miscarriage, preterm delivery, and PP depression (11, 12). Among autoimmune thyroid diseases, Graves’ disease is of particular relevance in pregnancy. In fact, Graves’ disease together with its therapy could affect maternal and fetal outcome; however, pregnancy by itself could change the presentation and course of Graves’ disease. This review is focused on the role of TSH receptor antibodies (TRAbs) that represent the hallmark of Graves’ disease and are able to influence, contemporarily and/or independently, both the maternal and the fetal thyroid function.

## Graves’ Disease in Pregnancy

**Figure 1:**
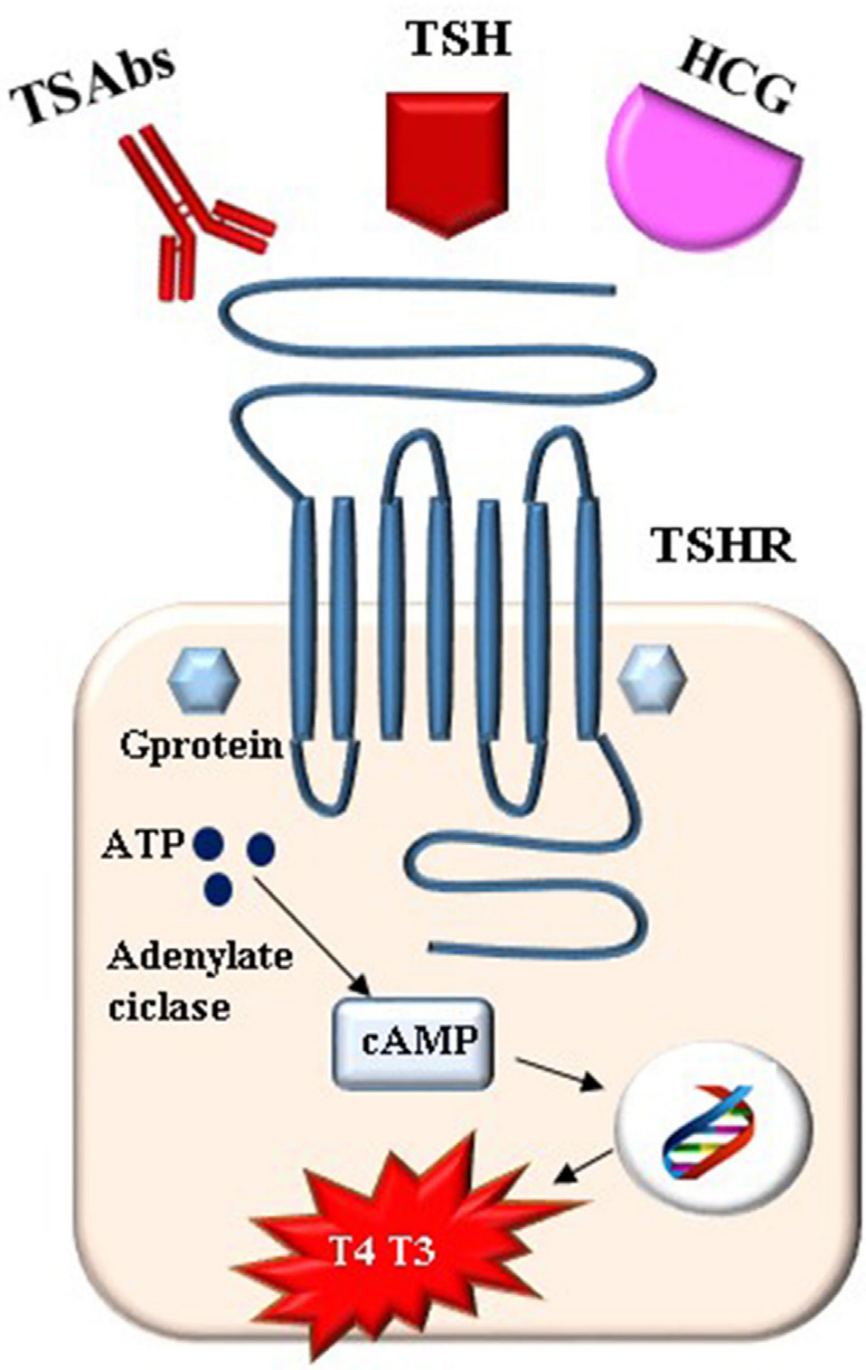
The stimulation of TSH receptor (TSHR) in pregnancy.

Graves’ disease occurs before pregnancy in 0.4–1% of women and in 0.2–0.4% during pregnancy, representing the most common cause (85%) of either overt or subclinical hyperthyroidism in women of reproductive age (11, 13). A more frequent and peculiar form of hyperthyroidism in pregnancy is the gestational transient thyrotoxicosis (GTT) whose prevalence in Europe is estimated between 2 and 3% with higher levels (5.5–11%) in Asia (2, 14, 15). GTT is defined as transient thyrotoxicosis caused by the stimulating effect the β-HCG has on the TSH receptor toward the end of the first trimester of gestation and is frequently associated with hyperemesis gravidarum and twin pregnancies. The prevalence of other causes of thyrotoxicosis in pregnancy (multinodular toxic goiter, toxic adenoma, subacute or silent thyroiditis, iodide-induced thyrotoxicosis, thyrotoxicosis factitia, hydatidiform mole, and hyperplacentosis) is negligible (2). In a population-based study in Denmark, which included 403,958 women, the incidence of hyperthyroidism (defined by redeemed prescription of antithyroid drugs (ATDs) and assumed to be Graves’ disease) was high early in pregnancy, declined during gestation and significantly increased at 7–9 months PP. Such a pattern was not observed for other autoimmune diseases (16). This observation acts as a clue as to the peculiar course of Graves’ disease in pregnancy. Different clinical scenarios can be observed in pregnant women: (1) stable active diseases receiving ATDs, (2) relapse in pregnancy after a ATDs course-induced remission, (3) *de novo* onset early in pregnancy, and (4) previous surgery or radioiodine treatment with persistence of TRAbs (Table 1). Women with a stable disease on ATDs could experience the worsening of hyperthyroidism early in pregnancy due to the additive thyroid-stimulating effect of HCG (Figure 1). In the same way, undiagnosed, subclinical Graves’ hyperthyroidism may become overt early in pregnancy. Early pregnancy relapse after ATDs withdrawal can be observed in women who have been treated for less than 6 months, or have ophthalmopathy or high levels of TRAbs (17). Later in pregnancy, Graves’ hyperthyroidism improves with remission in up to 30% of women by the middle of the third trimester and with relapse during the PP period (18). The main explanation for this course is the decrease of TRAbs due to the pregnancy-related immunosuppression/hemodilution. A contribution to the clinical improvement is the reduction of iodine pool and the increased binding capacity of TBG resulting in reduced free and active thyroid hormones.

**Table 1:** Clinical scenarios of Graves’ disease in pregnancy.

Stable Graves’ disease receiving antithyroid drugs (ATDs)
Relapsed Graves’ disease after an ATDs course
*De novo* onset of Graves’ disease in early pregnancy
History of Graves’ disease treated with radioiodine/surgery

### Diagnosis, Complications, and Therapy

The diagnosis of hyperthyroidism in pregnancy is straightforward in women with Graves’ disease already known before pregnancy since these women undergo pre-pregnancy counseling and monitoring at early stages. If this is not the case, the main diagnostic challenge is to identify signs and symptoms of thyrotoxicosis as well as to distinguish Graves’ disease from GTT. The symptoms vary depending on the degree of thyrotoxicosis. The clinical manifestations are often well pronounced in Graves’ disease while they may be absent or hidden in GTT. Some symptoms and signs may overlap with those typical of a hyperdynamic state of pregnancy, while others are more specific such as tachycardia, frequently exceeding 100 bpm, proximal muscle weakness and the failure to gain weight despite an increased appetite. The presence of goiter and/or of extra thyroidal manifestations, such as ophthalmopathy or dermopathy, is a clinical clue for Graves’ disease (2, 17). Clinical suspicion of hyperthyroidism needs to be confirmed by the finding of elevated serum FT4 concentrations and of suppressed serum TSH levels. The measurement of TRAbs is helpful in clarifying the etiology of thyrotoxicosis and a positive result strongly supports the diagnosis of Graves’ disease (9). Thyroid ultrasound and color Doppler could add clues showing a hypoechoic pattern and intense vascularity, radionuclide scintigraphy or radioiodine uptake are contraindicated during pregnancy. The measurement of TRAbs could have higher diagnostic value early in pregnancy given that their levels tend to decrease as pregnancy progress due to the physiological immunosuppression (2). The clinical significance of a positive TRAbs result goes beyond the diagnostic role for the mother since it is more relevant to predict fetal thyroid dysfunction. TRAbs cross the placenta and could cause goiter and hyperthyroidism in the fetus; therefore, Graves’ disease in pregnancy has long been recognized as an indisputable indication for TRAbs measurement (8, 9, 19). Several prospective and retrospective studies have highlighted that overt hyperthyroidism is associated with several adverse effects on pregnancy outcomes, which are directly related to the duration of thyrotoxicosis throughout a pregnancy. The most frequent complication is pregnancy-induced hypertension; the risk of eclampsia is five times higher in uncontrolled hyperthyroid women compared to controlled women and non-hyperthyroid pregnant women (20, 21). The overlap of hypertension to the left ventricular dysfunction induced by prolonged thyrotoxicosis could precipitate into congestive heart failure. Overt hyperthyroidism also increases the risk for intrauterine growth restriction, spontaneous preterm labor, preterm birth, gestational diabetes mellitus, cesarean delivery, and low birth weight infants. The highest risk for still birth (up to eight times) and low birth weight is observed in women with uncontrolled disease (22, 23). Subclinical hyperthyroidism is well tolerated from the mother and the fetus, while overt hyperthyroidism needs to be adequately treated in order to prevent obstetric and medical complications. The treatment of choice for overt hyperthyroidism in pregnant women is ATDs (8, 9, 13). Radioiodine treatment is contraindicated in pregnancy. Surgery is indicated in selected cases such as severe side effects of ATDs, or uncontrolled thyrotoxicosis despite high ATDs doses, and should be planned preferably in the second trimester of pregnancy to minimize the potential teratogenic effects of anesthetic agents. The drugs used are the propylthiouracil (PTU) and methimazole (MMI). PTU is generally preferred during the first trimester of pregnancy and then changed to MMI because of the risk of MMI-induced embryopathy, mainly aplasia cutis, esophageal, and choanal atresia (24–26) whose prevalence is reported higher than previously thought (27). The prevalence of the PTU-associated, but less severe, birth defects is not negligible (9, 28). After the first trimester, MMI is the preferred ATD because PTU has a greater risk of hepatotoxicity (9). The starting dose varies according to the extent of thyrotoxicosis and the equivalent potency MMI to PTU is 1:20 (9). It is worth remembering that both MMI and PTU cross the placenta (29) and tend to have greater effect on the fetal thyroid function compared to the mother. Therefore, ATD doses need to be tailored to correct the maternal thyrotoxicosis and to avoid fetal hypothyroidism, which is detrimental for the fetal brain development. This is achieved by using the minimum dose of ATDs to maintain the concentration of FT4 in the high values of the normal non-pregnant range irrespective of TSH levels whose normalization would require doses able to determine fetal hypothyroidism. In most cases due to the pregnancy-induced amelioration of Graves’ disease, the dose can be gradually reduced and ATDs even discontinued in third trimester especially in women with negative or decreasing TRAbs.

## TRAbs in Pregnancy

In pregnancy, as in the non-pregnant state, TRAbs are a hallmark of Graves’ disease. TSH receptor (TSHR), thyroglobulin (Tg), and thyroid peroxidase (TPO) are the immune targets of autoreactive T cells and autoantibodies in autoimmune thyroid disease, but while Tg and TPO autoantibodies are detected also in healthy subjects, anti-TRAbs can be found only in sera of most patients with Graves’ disease and in 10–15% of patients with Hashimoto’s thyroiditis (30). TRAbs are also unique among antithyroid autoantibodies having a key pathogenetic role in determining the hyperthyroidism and the extra thyroidal manifestation of Graves’ disease such as ophthalmopathy (31). It is worth to note that the term TRAbs means antibodies able to interact the TSH receptor, regardless of their action and of the method employed to detect them (32). The characterization of TRAbs has been the subject of research since the original description of long-acting thyroid stimulators in the fifties (33). Nowadays, it is known that TRAbs are able to influence thyroid function acting on the TSHR in different ways: stimulating (TSAbs), blocking (TBAbs), and without determining functional response with a neutral effect (N-TRAbs). TSAbs are the hallmark of Graves’ disease (31). TBAbs can also be observed in Graves’ disease being responsible for the evolution toward hypothyroidism in a small percentage of patients while they have a pathogenetic role in the atrophic form of Hashimoto’s thyroiditis (34). Switching between TBAb and TSAb (or *vice versa*) occurs, although rarely, in hypothyroid patients and in ATDs treated patients with Graves’ disease (35).

### TRAbs Assays

Given the pathogenetic and prognostic role of TRAbs in Graves’ disease, it is not surprising that research has made a strong effort in the development of methods to quantify and characterize TRAbs, which could be useful in the clinical management. Different assays for the detection of TRAbs have been available for more than 30 years over different generations of laboratory methods and great improvement in sensitivity and specificity have been achieved. The description of the different assay methods is beyond the scope of this review and is exhaustively detailed elsewhere (30, 31, 36–39). Briefly, two different methods can be distinguished: “receptor assays” and “bioassays.” Receptor assays measure TSH-binding inhibiting immunoglobulins (TBII) meaning that they detect serum autoantibodies by their capacity to compete for the binding of labeled TSH to an *in vitro* TSH receptor preparation. Three generations of TBII assay have been developed. The first-generation assays are competitive immunoassays in liquid phase and they detect the inhibition, by antibodies in the patient’s serum, of the binding of radio- or enzyme-labeled TSH to thyroid membrane extracts. The second generation assays are competitive immunoassays in solid phase, which use recombinant human TSHR or porcine TSHR. The third-generation assays are solid-phase competitive immunoassays based on the competition between antibodies in the patient’s serum and a human labeled thyroid-stimulating monoclonal antibody (M22) for the binding to TSHR. Increasing sensitivity and specificity has been achieved through the different generations of immunoassays and great progress has been made in the automation of assays. Overall the sensitivity and specificity of the second- and third-generation TRAbs assays are 86.5 and 97.4%, and 97 and 99.2%, respectively, with little difference between the types of immunoassay methods used (human or porcine receptor, manual or automated procedure) (39). The major limitation of receptor assays is that they are not able to evaluate functional properties of the TRAbs (i.e., they do not differentiate between TSAb and TBAb in serum samples). Therefore, they do not predict the phenotypes of Graves’ disease and a lack of correlation between TRAbs levels, measured using these assays, and the clinical and biochemical severity of the disease can be observed. Bioassays are functional tests that have the main advantage to detect the functional properties of TRAbs, i.e., stimulating (TSAbs) or blocking (TBAbs). This is accomplished by incubating the patient’s serum with cultured cells natively or artificially expressing TSHR (FRTL-5 or CHO cells) and then measuring the cyclic AMP production by the use of radioimmunoassay or by chemiluminescent assay. Similar to immunoassays, bioassays have gone through significant improvement from technically demanding methods to assays now available as commercial kits (39). A new Mc4 bioassay that measures only TSAbs, without interference of blocking TRAbs, is now available in commercial kit showing good sensitivity and specificity (40, 41). This assay selectively detects TSAbs because it is based on cells expressing a chimeric receptor that, compared to the wild-type, retains the main binding site of the TSAbs, but loses the main epitope recognized by TBAbs, which is replaced with the same receptor portion of the LH/hCG. On the other hand, a bioassay selectively detecting the TBAbs has been developed using a chimeric TSHR (42). Pregnancy entails several differences in interpretation, behavior, and in the role and significance of TRAbs compared to a non-pregnant state. The still point is the fact that during pregnancy, TRAbs readily cross the placenta. Therefore, potential effects on expectant mothers, as well as fetal thyroid function during pregnancy and, again, with neonatal and mother thyroid function in the PP are dealt with (43). Starting from methodological issues of TRAbs detection, the best TRAbs assay used in pregnancy should be the bioassay since the functional activity of the TRAbs is crucial, especially for the fetus (11). In fact, if the TRAbs are detected in a hyperthyroid pregnant women, they obviously have stimulating properties, this is not true in women who show detectable TRAbs levels but are no longer hyperthyroid having received definitive treatment for their disease (i.e., surgery or radioiodine). In these women, information on biological activity of the TRAbs is crucial to predict their effect on the fetus. Receptor assays and bioassays have a complementary role in pregnancy (36, 44).

### TRAbs Changes during Pregnancy

**Figure 2:**
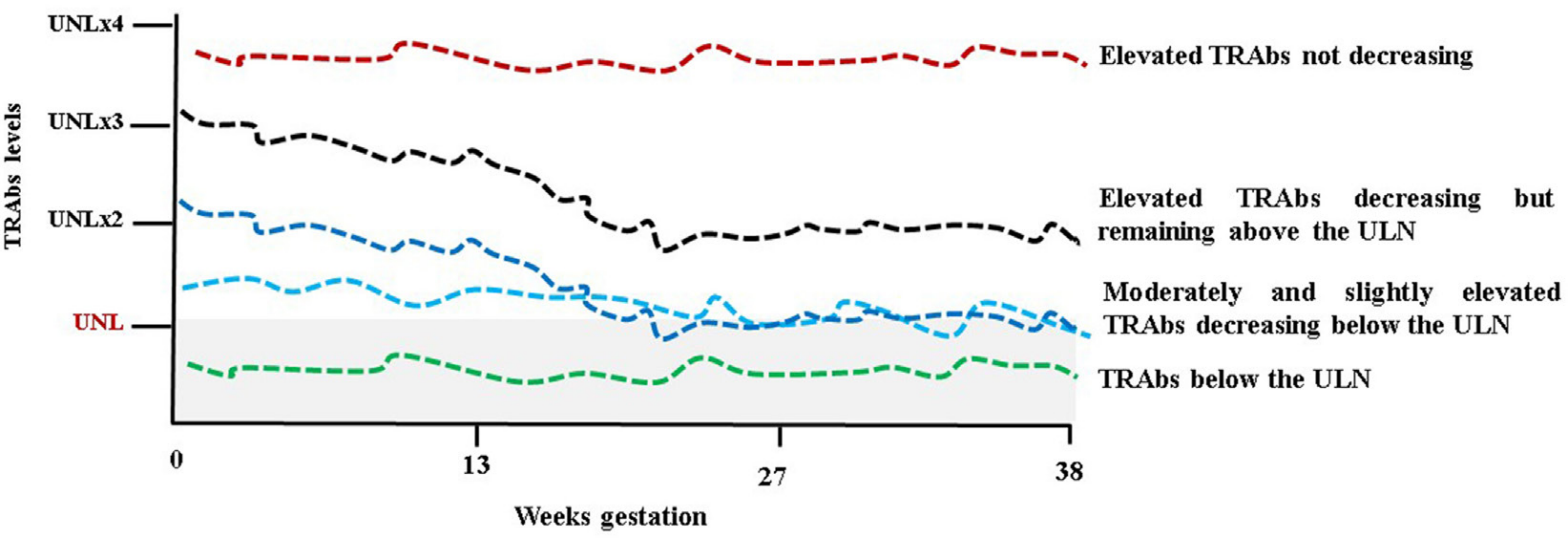
Schematic representation of TSH receptor antibodies (TRAbs) behavior during pregnancy. UNL, upper normal limit and (x) multiples. The gray shaded area represents the normal limit.

Regarding the behavior of the TRAbs, it is remarkable to note that due to the pregnancy-induced immunosuppression autoantibodies levels tend to decrease throughout pregnancy. The most typical scenario is that the TRAbs are detectable in the first trimester, but their levels decrease after 20 weeks of gestation becoming undetectable toward the term of pregnancy. This reflects the amelioration in thyrotoxicosis commonly observed. A study of 45 pregnant GD women (20 treated with ATDs throughout pregnancy and 20 in remission before pregnancy) showed a significant decrease in the TRAbs levels (measured by a first-generation immunoassay) with a significant rebound PP (45). In a study from Japan, the TRAbs levels were measured serially in 23 women from early to late pregnancy using four methods (first-, second-, and third-generation TBII assays and bioassay) and a decrease in the TRAbs, irrespective of the assay method used, was observed as pregnancy progressed (46). In a more recent study of 42 pregnant women, TRAbs levels (measured by second-generation TBII assay) decreased or remained stable in 86% of patients while rose in 14% (47). Nevertheless Graves’ disease’s course is variable in pregnancy as well as in a non-pregnant state and therefore there are women whose TRAbs, although at low levels, remain stable in pregnancy as well as women with more severe Graves’ disease with high levels of TRAbs not decreasing throughout gestation (Figure 2) (48). The disappearance of the TRAbs in pregnant women with Graves’ disease, who are euthyroid on a low dose of ATDs, supports the decision to reduce or withdraw medications in late pregnancy. In this scenario, in fact, fetal/neonatal hyperthyroidism is less likely in respect to an ATD-induced fetal hypothyroidism (9). TRAbs could persist for a varied amount of time after definitive therapy for Graves’ disease (radioiodine or surgery). It has been established that radioiodine therapy can lead to the worsening of autoimmunity with increasing TRAbs levels (49, 50). In a prospective randomized study, the TRAbs were serially measured in patients treated with ATDs, subtotal thyroidectomy, and radioiodine therapy. During ATDs treatment and after surgery, the TRAbs levels gradually decreased to reach the upper level of the normal reference interval for the assay after approximately 1 year and disappeared in 70–80% of the patients after 18 months. After radioiodine, an increase in the TRAbs was observed immediately after therapy with a maximal value at 3 months. Thereafter, levels slowly returned to pretreatment levels in 1 year, and continued slowly to decrease; however, average values were well above the normal reference throughout the 5 years with approximately 40% of patients still TRAbs-positive (51). In a recent study, a serial evaluation of TRAbs levels, measured by a quantitative third-generation assay, after total thyroidectomy showed that the TRAbs values decreased rapidly in most of the patients, especially within the early postoperative period (3 months). Nevertheless, the TRAbs half-life ranged from 3 months in patients with Graves’ disease not complicated with ophthalmopathy and not smoking, to 5 months in patients with ophthalmopathy or smoking and up to 1 year in patients with ophthalmopathy and smoking (52). The course of the TRAbs after surgery and/or radioiodine need to be kept in mind in order to estimate the time needed to achieve the maternal safe value in women planning pregnancy (53). It could be said that the TRAbs could persist beyond the suggested interval of 4–6 months to avoid conception for radioprotection and also beyond the time to reach stable euthyroidism after surgery (8, 9). As mentioned above, this is the only clinical situation where the bioactivity of the TRAbs may need to be known since their effects on the fetus cannot be predicted from the maternal thyroid function (11). Attention must be given to these women since isolated fetal hyperthyroidism could develop despite maternal euthyroidism or adequately replaced hypothyroidism. On the other hand, fetal hypothyroidism could also develop if autoantibodies have a blocking activity (9). Apart from a “quantitative” change of the TRAbs, a “qualitative” change, i.e., a variation of their functional properties has been evidenced in pregnancy. Indeed switching between TBAbs and TSAbs (or *vice versa*) occurs, although unusually, in patients during L-T4 replacement therapy or ATDs treatment for Graves’ disease (35). Changes from stimulating to blocking activity of TRAbs could contribute to the improvement/remission of thyrotoxicosis in pregnancy. In a study that included 15 pregnant women with Graves’ disease receiving no or a low dose of ATDs and 14 healthy pregnant women, sera were tested for TRAbs, by first-generation receptor immunoassay (TBII), and for TSAbs and TBAbs by bioassays. The healthy pregnant women were all negative for TSAbs, TBII, and TBAbs. In pregnant women with Graves’ disease, the TSAbs decreased significantly during pregnancy, and the TBAbs significantly increased. The TBII fluctuated and showed no correlation to the TSAb activity (54). Another study brought the same conclusion. In the sera of 13 pregnant women with Graves’ disease during pregnancy and PP TBII, TSAbs and TBAbs (the last two detected by assays employing chimeric receptors) were measured. As pregnancy advanced, the TSAbs decreased and the TBAbs increased while the TBII, although fluctuating, did not change significantly. The TBAbs appeared during pregnancy also in women who were negative for TSAb (55). These findings were not confirmed in a study of six patients with Graves’ disease receiving no or a low dose of ATDs. During pregnancy, the TBII and the TSAbs decreased gradually but increased after delivery. The TBAbs were lower than the cutoff value in early pregnancy, and further significantly decreased in four patients during pregnancy (56).

## TRAbs and Fetal–Neonatal Thyroid Function

**Figure 3:**
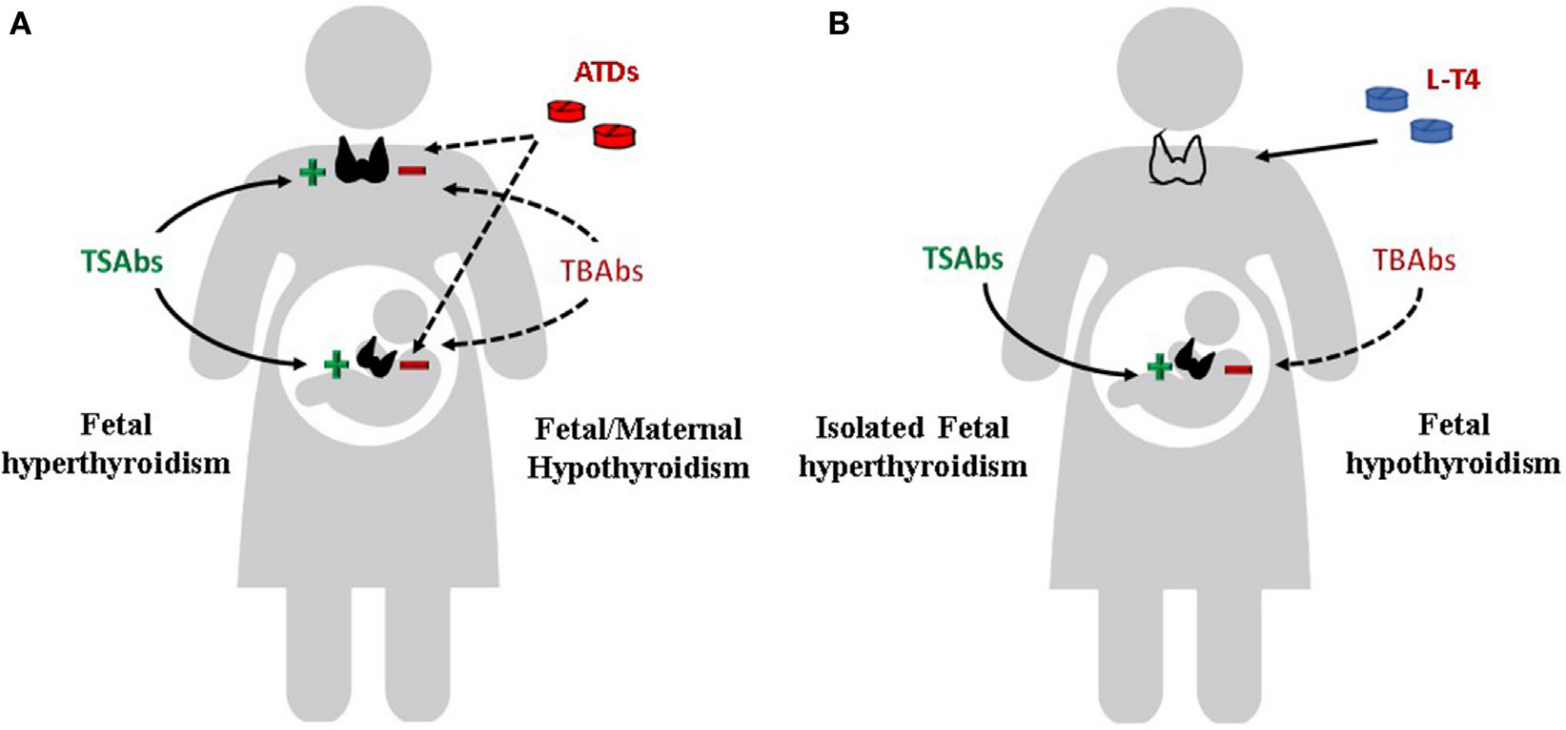
Effects of TSH receptor antibodies and antithyroid drugs (ATDs) on maternal and fetal thyroid function. **(A)** Maternal and fetal thyroid are stimulated by TSAbs (continue line) and inhibited by ATDs and TBAbs (dotted line). If TBAbs are present, fetal as well as maternal hypothyroidism can occur. **(B)** Maternal hypothyroidism on L-T4 replacement after radioiodine therapy or thyroidectomy for Graves’ disease. Isolated fetal hyperthyroidism can occur. If TBAbs are present, fetal hypothyroidism can also occur.

Apart from the clinical significance of the TRAbs in determining the course of Graves’ disease in pregnant women, a more relevant role is attributed to the autoantibodies in affecting the fetal and neonatal thyroid function. The fetal/neonatal thyroid dysfunction is the ideal human *in vivo* experimental system for the evaluation of the TRAbs (Figure 3). TRAbs easily cross the placenta from the first weeks of gestation. However, placental permeability is low early in pregnancy and increases progressively. The fetal thyroid becomes responsive to the TSH and to the TRAbs at around week 20 of gestation.

### Fetal and Neonatal Hyperthyroidism

Fetal hyperthyroidism, which is the more common and expected dysfunction, develops usually at around 26 weeks, or as early as 18 weeks in severe cases (57). The prevalence of fetal hyperthyroidism is difficult to establish and several case reports have been published (58). Untreated maternal Graves’ disease can lead to severe fetal hyperthyroidism and this could explain perinatal mortality of 20–45% observed before the introduction of ATDs (59, 60). Alternatively, mild fetal hyperthyroidism may not be noticed. Fetal hyperthyroidism is almost invariably followed by neonatal hyperthyroidism whose prevalence seems to be better established. Overt neonatal hyperthyroidism is reported in 1–5% of neonates born to mothers with Graves’ disease (58, 61, 62). Fetal/neonatal hyperthyroidism is associated with the maternal thyroid condition (serum FT4 levels, dose of ATDs required to achieve adequate euthyroidism) and with the serum TRAbs levels (63). Several studies were aimed to establish a threshold of maternal TRAbs levels that could define the risk of fetal/neonatal hyperthyroidism. Using first-generation TRAbs assay, an increased risk of fetal/neonatal hyperthyroidism was reported if the maternal level of TBII was over 40 IU/l (normal range, <10 IU/l) or over 50% (normal range, <10–15%) in the third trimester of pregnancy (19, 64–66). In a study including 62 pregnant women with Graves’ disease, TRAbs were measured by four immunoassays: first generation, second generation using porcine TSHR, second generation using human recombinant TSHR, and third generation. The first generation assay cutoff (>50%) correlated with the equivalent for second (>10 IU/L) and third (>75%) generation assay cutoff in predicting the risk of fetal–neonatal dysfunction. However, third-generation assay identified additional high-risk women whose first-generation TRAbs were below 50%. In the same study TSAbs were assayed in 20 mothers, 4 of them, having high TSAbs values ranging from 412 to 1,584%, gave birth to infants with hyperthyroidism; this was not observed in newborns born to mothers whose TSAbs were below 400%, regardless of the TBII value (46). More recently, a study of 47 neonates born to 42 mothers with measurable levels of TBII during pregnancy (assayed by second-generation immunoassay) showed that all the 9 hyperthyroid neonates were born to mothers with TRAbs values above 5 IU/L in the second trimester of pregnancy. A TBII value over 5 IU/l (which is 3.3 times the detection level of the method) during the second and third trimester of pregnancy predicted the neonatal hyperthyroidism with a sensitivity of 100% and a specificity of only 43%. Measurement of the TSAbs by bioassay showed that no mother with TSAbs below 400% gave birth to a hyperthyroid neonate (47). In summary, fetal/neonatal hyperthyroidism can be predicted by maternal TRAbs levels. According to guidelines, in clinical practice fetal surveillance is recommended in women with TRAbs levels exceeding three times the upper limit of normal at any time during gestation (9). Therefore, determination of TRAbs to establish the risk of fetal/neonatal hyperthyroidism is a recommendation shared by the various guidelines that have been published (8, 9, 19, 67, 68) (Table 2). At-risk pregnancies need to be monitored carefully, with repeated ultrasound examinations from 20 weeks of gestation onward, to screen for goiter and ultrasound findings of fetal thyroid dysfunction (58). Indeed the maternal history, the thyroid functional status, the TRAbs levels, and ultrasound parameters are the diagnostic clues for fetal hyperthyroidism. Thyroid gland enlargement is the first sign that suggests fetal hyperthyroidism and precedes fetal tachycardia (fetal heart rate >160/min). The fetal thyroid size needs to be determined by using normative data according to the gestational age (69). It is worth remembering that fetal goiter can also be observed in hypothyroid fetuses as a consequence of the maternal ATDs transfer. Peripheral thyroid vascularization, instead of diffuse increased blood flow, delayed bone maturation, and fetal heart rate <160/min, favors the diagnosis of fetal hypothyroidisms. Other ultrasound findings of hyperthyroidism are accelerated bone maturation (i.e., distal femoral center seen before 32 weeks) and intrauterine growth retardation. Fetal growth should be followed by standard gestational age sonographic parameters, in particular the abdominal circumference, since hyperthyroid fetuses are thin. The sensitivity and specificity of fetal thyroid ultrasound at 32 weeks for the diagnosis of clinically relevant fetal thyroid dysfunction are reported to be 92 and 100%, respectively, and could replace invasive and hazardous examinations, such as fetal blood collection or amniotic fluid sampling (48, 70). Early diagnosis and treatment of fetal hyperthyroidism are crucial to prevent death *in utero*, premature delivery, or congestive heart failure. Fortunately, the ATDs used to treat maternal hyperthyroidism cross the placenta, thus controlling fetal hyperthyroidism. The ATDs tend to overtreat the fetus and therefore the dose of drug given to the mother needs to be as low as possible (Figure 3). The monitoring of the maternal thyroid function and fetal ultrasound are the clues for the management of Graves’ disease in pregnancy. A peculiar form of fetal isolated hyperthyroidism is that observed in euthyroid or hypothyroid pregnant women previously treated with surgery and/or radioiodine for GD since, as discussed above, TRAb could persist for years. Fetal/neonatal hyperthyroidism has been described in two consecutive pregnancies in a woman treated with surgery 10 years before the first pregnancy (71) and in up to 11% of women treated with radioiodine whose levels of TRAbs did not decrease during gestation regardless of the time from the radioiodine treatment to conception (72). In these patients, fetal thyroid stimulation can occur, despite maternal euthyroidism or levothyroxine replaced hypothyroidism. These are the only women who should receive block and replacement therapy in pregnancy, i.e., ATDs to treat the fetal hyperthyroidism, and levothyroxine to keep the mother’s euthyroidism remembering that the placental transfer of ATDs is greater than that of levothyroxine. In a review, 11 published reports involving 13 pregnancies, the ATDs treatment of mothers whose TRAbs levels were >5-fold above normal, resulted in 13 live births while in previous pregnancies 6 serious complications (miscarriages, stillborn, or infant deaths) had been observed (73). Neonatal hyperthyroidism is suspected in newborns presenting tachycardia, hyperexcitability, and poor weight gain. Goiter, eyelid retraction and/or exophthalmos, small anterior fontanel are additional clinical clues. Congestive heart failure is one of the major immediate causes of morbidity. However, long-term complications such as craniosynostosis, microcephaly, and psychomotor disabilities may occur in severely affected newborns (58). It is remarkable to notice that signs of hyperthyroidism may not become apparent until 2–5 days in newborns to mothers on ATDs. This is the time necessary for the ATDs to be cleared from the newborn circulation (70). Hyperthyroidism is transient and persists as long as the TRAbs become undetectable. The half-life of the TRAbs is estimated to be 2–3 weeks (74–76). Duration of treatment of infants with ATDs is most commonly 1–2 months and only exceptionally longer (47, 77). A correlation has been found between maternal TRAbs levels and neonatal hyperthyroidism. In a study of 172 pregnant women with Graves’ disease neonatal hyperthyroidism developed in 6.5% of infants, most of them were born to mothers whose TRAbs levels were 30% or more (i.e., 2–5 times above the normal range) at delivery (61). In a study of 29 women with a history of Graves’ disease and positive TRAbs, neonatal thyrotoxicosis developed in 17%. A TRAbs level threshold of 5UI predicted neonatal thyrotoxicosis with a sensitivity of 100%, specificity of 76.0%, positive predictive value of 40.0%, and negative predictive value of 100% (78). In a more recent study of 68 neonates born to mothers with GD, none of the infants born to TRAbs-negative mothers developed neonatal hyperthyroidism. 73% of infants born to TRAbs-positive mothers had positive TRAbs on cord blood assays, and 30% of these developed neonatal hyperthyroidism. All hyperthyroid neonates had cord blood levels of TRAbs greater than two times the upper normal level. A correlation was found between the TRAbs cord blood levels and the maternal serum TRAbs levels at term, thus confirming that the latter are a good predictor of neonatal hyperthyroidism. This was not the same for FT4 whose cord blood levels reflected fetal rather than neonatal thyroid function. The FT4 needs be revaluated on day 3–5 to establish thyrotoxicosis and the need for treatment (79).

**Table 2:** Indications and timing for TSH receptor antibody (TRAb) assays in pregnancy according to guidelines.

Society (reference)	Indication for TRAbs assay	Timing	TRAbs level at risk for fetal hyperthyroidism
ETA 1998 (19)	Euthyroid pregnant woman (with/without thyroid hormone substitution therapy) who has previously received radioiodine therapy or undergone thyroid surgery for Graves’ disease	Early in pregnancy and in the last trimester if antibodies are present	40 U/l

Endocrine Society 2007 (67)	Current Graves’ disease, history of Graves’ disease and treatment with ^131^I or thyroidectomy, previous neonate with Graves’ disease	Before pregnancy or by the end of the second trimester	

Endocrine Society 2012 (8)	Current Graves’ disease; history of Graves’ disease and treatment with 1^31^I or thyroidectomy before pregnancy; previous neonate with Graves’ disease; previously elevated TRAb	Week 22	2- to 3-fold the normal level

ATA 2011 (68)	Past or present history of Graves’ disease	Weeks 20–24	>3 times the upper limit of normal

ATA 2017 (9)	Past history of Graves’ disease treated with ablation (radioiodine or surgery)	Early in pregnancy repeat determination at weeks 18–22	>3 times the upper limit of normal
	Patient on antithyroid drugs (ATDs) for treatment of Graves’ hyperthyroidism when pregnancy is confirmed	Early in pregnancy	
	Patient requires treatment with ATDs for Graves’ disease through mid pregnancy	Repeat determination at weeks 18–22
	Elevated TRAb at weeks 18–22 or the mother is taking ATD in the third trimester	Repeat determination at weeks 30–34

### Fetal and Neonatal Hypothyroidism

As previously mentioned, sometimes the TRAbs have a blocking effect on the TSH receptor, thus inducing fetal and neonatal hypothyroidism (Figure 3). Measurement of TBAbs by a bioassay in dried neonatal blood specimens obtained from 788 neonates identified as congenital hypothyroidism at the neonatal screening program in US demonstrated potent TSHR-blocking activity in 11 cases. The 11 babies were born to 9 mothers, all of whom were receiving thyroid replacement because of autoimmune hypothyroidism, and 3 had been treated initially for Graves’ disease. TPO antibodies, although detectable in all mothers, did not predict the neonatal thyroid dysfunction, while the presence of TBAbs was confirmed in the serum of eight mothers: all newborns had transient congenital hypothyroidism. The author estimated the prevalence of TBAbs-induced congenital hypothyroidism in the order of 1 in 180,000, or about 2% of all cases (80). In a large series of newborns screened for congenital hypothyroidism in Wales (375 cases identified over 966,969 infants screened), 6 (1.6%) were found to have transient congenital hypothyroidism due to maternal TBAbs. All the mothers were hypothyroid on levothyroxine replacement therapy or were diagnosed with hypothyroidism after the reported elevation of TSH in their infants (81). The presence of TBAbs has been advocated to explain the delayed onset of neonatal hyperthyroidism in newborns to mother with Graves’ disease harboring both stimulating and blocking antibodies (82). In this situation, it can be hypothesized that differences in the receptor affinity, as well as in the clearance rate of the two populations of antibodies, determine the clinical course of thyroid dysfunction in the neonate (35).

## TRAbs in the Postpartum

During the PP a rebound reaction to the pregnancy-associated immunosuppression is observed and this explains the aggravation of autoimmune diseases during the puerperium. The levels of TRAbs could increase and women who experienced remission during late pregnancy, as well as women who were in remission after the ATDs course before pregnancy, could experience relapse in the PP. After ATDs withdrawal, relapse of Graves’ hyperthyroidism was observed in 84% of women who had further pregnancies compared to 56% of women who did not remain pregnant. The number of pregnancies after ATDs cessation was significantly correlated with the risk of relapse. The relapse of Graves’ hyperthyroidism occurred between 4 and 8 months after delivery (83). On the other hand, *de novo* onset of Graves’ disease after pregnancy has been reported in 7–8% (84). Few studies have focused on establishing a predictive role of the TRAbs positivity early in pregnancy for postpartum onset of Graves’ thyrotoxicosis. In 71 women with positive antithyroid microsomal antibody (MCAb), 10% showed positive TRAbs (both TBII and TSAb) in early pregnancy, although without any thyroid dysfunction; 71% of them developed Graves’ disease PP, none of the TSAbs-negative subjects developed Graves’ thyrotoxicosis. Various types of thyroid dysfunction as a result of postpartum autoimmune thyroiditis were found in 62% of MCAb-positive women (85). In a further study of 38 pregnant women who were positive for TPOAb, 10% were positive for TSAbs measured by a sensitive bioassay. PP Graves’ hyperthyroidism developed in 50% of TSAbs-positive women. These findings indicate that the third-generation TRAbs assay was not useful; however, a sensitive TSAbs bioassay was moderately useful for predicting the PP onset of Graves’ hyperthyroidism (86). Apart from the possible role played by the TRAbs assay in early pregnancy in predicting the risk of developing the disease in the puerperium, antibody testing plays an important role in clinical practice to differentiate Graves’ hyperthyroidism from thyrotoxic phase of postpartum thyroid dysfunction (PPTD). The PTDD occurs in approximately 5–10% of women in the general population within 1 year of delivery and that is significantly higher than the prevalence of Graves’ disease in childbearing age. A differential diagnosis is essential given the two conditions differ significantly in the course as well as in the treatment. In a series of 42 women developing PP thyrotoxicosis, 86% had PPDT and 24% had Graves’ disease. TRAbs measured with third-generation receptor assay were positive in all patients with Graves’ disease and negative in all patients with the PTTD; the latter also showing low thyroid blood flow measured quantitatively by color flow Doppler ultrasonography. The PPTD occurred earlier (3 months or less earlier after delivery), while Graves’ disease developed at 6 months or later (87). In another study, the second-generation assay for TRAbs was useful to differentiate the relapse of Graves’ thyrotoxicosis from development of painless thyroiditis in patients who seemed to be in remission after ATDs treatment for Graves’ disease. 85.7% of 14 patients with a relapse of Graves’ thyrotoxicosis were positive for TRAbs, and 91.7% of 12 patients who developed painless thyroiditis after ATDs treatment for Graves’ disease were negative for TRAbs (88). The clinical relevance of these observations is that in women with history of Graves’ disease, thyroid function monitoring and TRAbs measurement are needed in the PP, irrespective of the course the disease takes during pregnancy. Beyond the role of the changes in the autoimmune response occurring during gestation and in the PP, it has to be highlight that pregnancy and delivery have to be considered stressful events, which could have, on their own, a causative role in the onset, relapse or exacerbation of Graves’ disease. In a paradigmatic case report, a combination of stressful life events and pregnancy is reported. In a young woman, the onset of Graves’ disease shortly followed an emotional stress. The woman was treated with ATDs and experienced exacerbation of hyperthyroidism during her first pregnancy and 9 months after her first delivery. In both occasions, a stressful life event was retraced in her history (89). It has been reported that there exist patients with Graves’ disease in whom onset, exacerbation or relapse of hyperthyroidism are systematically preceded by at least one stressful event (90). Very recently, in these group of patients, HLA typing has demonstrated that both HLA class I and class II molecules are associated with stress-triggered Graves’ with certain HLA alleles and loci predisposing, while others protecting from stress-related Graves’ disease (91).

## Conclusion

Thyroid diseases in pregnancy affect the physiological mechanisms that allow the thyroid function to be adequate both for maternal and fetal requirements. Thyroid autoimmune diseases are the most common cause of thyroid dysfunction in childbearing women and thyroid autoantibodies are associated with several adverse maternal and fetal outcomes. TRAbs, which are the pathogenetic hallmark of Graves’ disease, present peculiar challenges in pregnancy. In fact, unlike Tg and TPO autoantibodies, they can directly affect contemporarily and/or independently, fetal as well as maternal thyroid function. On the other hand, pregnancy-related immunosuppression in most cases reduces the maternal levels of antibodies. Information about the TRAbs status (presence, levels), on their behavior (changes after radioiodine therapy or surgery and during gestation and PP) and on their multifaceted properties (stimulating or blocking activity) are essential for the preconceptional counseling as well as for the therapy of Graves’ disease during gestation and in the PP. This information is also crucial for the prediction and for the management of fetal thyroid dysfunction. The preservation of maternal and fetal euthyroidism is the challenge of the management of Graves’ disease in pregnancy.

## Author Contributions

IB: substantial contributions to the conception and design of the work; reviewing the literature; drafting the work; final approval of the version to be published; and agreement to be accountable for all aspects of the work in ensuring that questions related to the accuracy or integrity of any part of the work are appropriately investigated and resolved. CG: substantial contributions to the design of the work; revising the work critically for important intellectual content; final approval of the version to be published; and agreement to be accountable for all aspects of the work in ensuring that questions related to the accuracy or integrity of any part of the work are appropriately investigated and resolved. GN: substantial contributions to the conception of the work; revising it critically for intellectual content; final approval of the version to be published; and agreement to be accountable for all aspects of the work.

## Conflict of Interest Statement

The authors declare that the research was conducted in the absence of any commercial or financial relationships that could be construed as a potential conflict of interest.
